# Harnessing the Hepatoprotective and Nephroprotective Potential of *Nigella sativa* Fractions via per os Administration in CCl_4_-Intoxicated Wistar Rats: A Mixed Approach

**DOI:** 10.3390/ph18081147

**Published:** 2025-08-01

**Authors:** Mohammed Dalli, Nour Elhouda Daoudi, Salah-eddine Azizi, Mohammed Roubi, Ilyass Alami Merrouni, Faiza Souna, Mohammed Choukri, Bonglee Kim, Nadia Gseyra

**Affiliations:** 1 Higher Institute of Nursing Professions and Health Techniques, Oujda 60000, Morocco; nourelhoudada95@gmail.com (N.E.D.); azizi.salah-eddine@ump.ac.ma (S.-e.A.); alami.ilyass.90@gmail.com (I.A.M.); faizasouna@hotmail.fr (F.S.); 2 Laboratory of Bioresources, Biotechnology, Ethnopharmacology and Health, Faculty of Sciences, University Mohammed the First, Oujda 60000, Morocco; mohammed.roubi1@ump.ac.ma (M.R.); ngseyra@gmail.com (N.G.); 3 Central Laboratory, Biochemistry Department of Mohammed VI University Hospital Center, Oujda 60000, Morocco; choukrimohammed@hotmail.com; 4 College of Korean Medicine, Kyung Hee University, Hoegidong Dongdaemungu, Seoul 05253, Republic of Korea; 5 Korean Medicine-Based Drug Repositioning Cancer Research Center, College of Korean Medicine, Kyung Hee University, Hoegidong Dongdaemungu, Seoul 05253, Republic of Korea

**Keywords:** *Nigella sativa*, liver protection, kidney protection, molecular docking, ADMET

## Abstract

**Background:** *Nigella sativa*, known as black cumin, is traditionally used to treat various illnesses. **Objective:** The current study aims to investigate the potential hepatoprotective and nephroprotective effect of black cumin fractions via per os route in CCl_4_-intoxicated *Wistar* rats. This study used a computational approach to assess the interaction of bioactive compounds with key proteins (CYP P450 3E1, TNF-α, and Cox-2). **Methods:** *Wistar* rats were treated with CCl_4_ to induce liver injury and with different *Nigella sativa* fractions (250 mg/Kg) or Sylimarin (50 mg/Kg). Liver and kidney functions were assessed through biochemical markers, hepatic glycogen, malondialdehyde levels, molecular docking, and ADMET analysis to evaluate drug-likeliness. **Results:** The results revealed that intoxication with CCl_4_ induced an elevation in different liver and kidney biochemical parameters such as (ALT, AST, creatinine, urea...) indicating kidney and hepatic toxicity. However, treatment with different *Nigella sativa* fractions showed a significant improvement in animal body weight and significant amelioration of biochemical markers indicating a protective potential of these fractions against CCl_4_-induced intoxication. Furthermore, the molecular docking approach demonstrated high binding affinity with the target proteins. **Conclusions:** These current findings shed light on the therapeutic potential of *Nigella sativa* fractions as a promising protective agent of the liver and kidney against CCl_4_ intoxication.

## 1. Introduction

The liver is considered a metabolic hub in our body. It plays important physiological roles, including macronutrient metabolism, lipid and cholesterol homeostasis, immune system support, endocrine regulation of growth signaling pathways, blood volume regulation, and the breakdown of xenobiotic substances, including many modern drugs [1]. The liver oxidizes lipids but can also package excess lipids for secretion and storage in other tissues, such as adipose tissue. Also, it is a major player in protein and amino acid metabolism as it is responsible for the majority of proteins secreted into the blood, the conversion of amino acids into energy, and the elimination of nitrogenous waste from protein breakdown in the form of urea [2]. Cirrhosis and liver failure can develop as a result of this organ’s dysfunction [3]. Other factors that can impair liver function include excessive alcohol consumption, autoimmune problems, and microbial infections [4,5]. Also, the kidney plays an essential role in homeostasis, particularly through its ability to eliminate metabolic waste and maintain hydro-electrolytic and blood pressure balance. The kidney is exposed to significant risks, as most elements are eliminated by this organ. Drug-induced nephropathies typically result in the onset of acute kidney diseases that persist only during the treatment period [6,7]. However, some chronic kidney diseases can progress to end-stage renal failure. Additionally, non-steroidal anti-inflammatory drugs such as aspirin and paracetamol, as well as aminoglycosides and carbon tetrachloride (CCl_4_), can cause severe damage to this organ [8,9,10].

*Nigella sativa* (*NS*), commonly known as black cumin, is a plant species belonging to the Ranunculaceae family. It has been largely used in traditional medicine for its availability, low cost, and large therapeutic benefits [11,12]. Also, it has been stated that *NS* seeds have been used in a range of Persian dishes, including yogurt, pickles, sauces, and salads, as well as utilized as a food preservative and spice [13]. In fact, this plant has been used worldwide to treat multiple illnesses like asthma, inflammation, cough, eczema, and flu-like conditions. Also, *NS* seeds are used as a diuretic, carminative, and dewormer [14,15,16]. In Morocco, particularly, this plant is utilized for addressing various health issues including allergies, heart conditions, high blood pressure, skin scars, dermatitis, abdominal discomfort, stomachaches, vomiting, osteoarthritis, cancers, and rheumatic pain [17,18,19]. Moreover, *NS* is traditionally used in Morocco for kidney detoxification and to treat several liver diseases [20,21]. Despite this, there remains a lack of comprehensive scientific validation of these traditional uses, especially in the context of modern therapeutic applications. Recent investigations conducted in the field have uncovered that *NS* is endowed with abundant bioactive compounds such as phenolic acids, flavonoids, and alkaloids. Moreover, performed investigations have indicated the numerous health advantages of this panacea, demonstrating its immunomodulatory [22], anti-inflammatory [23], antimicrobial [23,24,25], antidiabetic [26], and antihypertensive properties [27]. Studies by Daba and Abdel-Rahman (1998) found that Thymoquinine (TQ), an active ingredient in *NS* seeds, has anti-inflammatory and antioxidant properties [28]. It also protects hepatocytes from apoptosis by balancing pro-apoptotic (like Bax) and anti-apoptotic (like Bcl-2) proteins. Furthermore, TQ stops the development of liver fibrosis by suppressing transforming growth factor-beta 1 (TGF-β1), a key mediator of fibrogenesis, and inhibiting collagen deposition [29], suggesting potentially effective treatment for fibrotic liver diseases. Additionally, *NS* showed an improvement in hepatic detoxification via modification of cytochrome P450 enzymes [30]. Similarly, NS was beneficial for the treatment of non-alcoholic fatty liver disease (NAFLD) [31].

These preclinical results are corroborated by clinical trials. In fact, *NS* (500 mg/day) decreased viral load and improved ALT levels in 48% of participants after three months, according to a randomized study (n = 75) on hepatitis C patients [32].

Furthermore, various experimental models have demonstrated these protective effects of *NS*. For example, 4 mL/kg of *NS* oil or 500 mg/kg extracts have shown protective effects against CCl_4_-induced liver damage and alcohol-induced hepatotoxicity in animal models [33,34]. However, these studies have only focused on crude extracts or fixed oils, and fewer have worked on isolated specific fractions to identify which components contribute most significantly to these protective effects. Additionally, limited studies have systematically investigated the nephroprotective and hepatoprotective effects of *NS* bioactive fractions using a combined in vivo and in silico approach, leaving a gap in understanding the detailed mechanisms of action, as well as the pharmacokinetic potential of its compounds.

The primary objective of this study is to evaluate the hepatoprotective and nephroprotective effects of *Nigella sativa* fractions in a CCl_4_-intoxicated Wistar rat model. Carbon tetrachloride (CCl4) was used as an established model of hepatotoxicity and nephrotoxicity due to its well-known ability to induce oxidative stress and organ damage by generating free radicals. This makes it an ideal agent for studying the protective effects of potential therapeutic compounds. To assess the efficacy of *Nigella sativa* fractions in mitigating CCl_4_-induced liver and kidney damage, a comprehensive panel of specific markers, including malondialdehyde (MDA), serum levels of liver enzymes (ALT, AST, ALP), and kidney function markers (creatinine, urea), was measured using biochemical assays in an Architect ci8200 analyzer. To ensure a better understanding of the results, the molecular docking approach was adopted in order to study the interaction mechanisms between the bioactive compounds identified in each fraction and the active sites of key target proteins involved in inflammation and oxidative stress pathways, such as NF-κB and TNF-α, and also in CCl_4_ metabolization, P450 3E1. Finally, pharmacokinetic properties, including absorption, distribution, metabolism, and excretion (ADME) profiles, were analyzed to evaluate the drug-likeness and therapeutic potential of these bioactive compounds.

## 2. Results

As reported in our previous study [35], *Nigella sativa* fractions were found to be rich in different bioactive compounds [35]. It also reported the presence of different compounds in different portions. In fact, catechin was found to be the abundant compound in the ethanolic fraction with a portion of 89.03 ± 0.011 mg/100 g of DW, followed by rutin (6.46 ± 0.004 mg/100 g of DW). Meanwhile, methanolic fraction was characterized by a high abundance of rutin (21.07 ± 0.105 mg/100 g DW), followed by gallic acid (19.92 ± 0.015 mg/100 g DW), catechin (13.79 ± 0.053 mg/100 g DW), and vanillic acid by a quantity of 4.36 ± 0.029 mg/100 g DW. However, the aqueous fraction of *NS* was endowed by the presence of a high amount of salicylic acid 32.26 ± 0.094 mg/100 g DW, followed by rutin, catechin, vanillic acid, and gallic acid [35]. On the other hand, the CG-MS analyzed the methyl ester n-hexane fraction, which indicated the presence of several compounds with different abundance percentages. The most abundant compound was linoleic acid, followed by palmitic and stearic acids. It also noted the presence of thymoquinone (8.70%) and carvacrol (3.03%) [35].

### 2.1. Per os Administration of Nigella Sativa Fractions on Rats’ Weight

The results depicted in Table 1 below show the *per os* administration effect of the different fractions of black cumin at a dose of 250 mg/kg versus intraperitoneal injection (*i.p*) of CCl_4_ at a dose of 1 mL/kg/week. The results showed that there was no significant change between the weights of animals in the different groups compared with the control group prior to CCl_4_ injection (*p* > 0.05) on the 0th and 7th days. On the 9th day, a highly significant difference was observed between the control and CCl_4_ groups (*p* < 0.0001). Similarly, there was a significant difference for the group treated with the methanolic fraction (*p* < 0.05) and a very highly significant difference after treatment with the n-hexane fraction (*p* < 0.0001) at a dose of 250 mg/kg, in comparison with the CCl_4_ group. There was no significant difference between the latter group and the others (*p* > 0.05).

On the 12th day, the comparison between the weight of the control group and that of the CCl_4_ group showed a highly significant difference (*p* < 0.0001). The comparison between the CCl_4_ injection group (1 mL/kg/week) and the other groups showed a significant difference with the group treated with Sylimarin (50 mg/kg) (*p* < 0.05), a highly significant difference with the group treated with the methanolic fraction (*p* < 0.001), and a very highly significant difference with the groups treated with the ethanolic and n-hexane fractions (*p* < 0.0001). Also, no significant difference was observed in the aqueous fraction-treated group (*p* > 0.05) (Table 1). Finally, on the 15th day, a highly significant difference persisted between the control and CCl_4_ groups (*p* < 0.0001). In addition, there was no significant difference between the methanolic fraction and Sylimarin-treated groups, compared with the CCl_4_ group (*p* > 0.05). However, there is a highly statistically significant difference in the group treated with the aqueous fraction (*p* < 0.01), the ethanolic fraction (*p* < 0.001), and the n-hexane fraction (*p* < 0.0001) compared with the CCl_4_ group (Table 1).

### 2.2. Black Cumin Effect on the Variation in Liver and Kidney Weight Ratios

The results reported in Table 2 below show the liver ratio and the right and left kidney ratios.

The results indicated a significant difference between the control group and the CCl_4_-intoxicated group (*p* < 0.05). However, no significant difference was observed between the CCl_4_ group and the groups treated with NS fractions when compared to the control. Additionally, there was no significant difference in the right and left kidney ratios across all groups (*p* > 0.05) (Table 2).

### 2.3. Per os Administration of Different Nigella Sativa Fractions on Water Consumption and Urine Volume in CCl_4_-Intoxicated Rats

The results tabulated in Table 3 summarize water consumption and urine volume measured at the end of the experiment. There was a highly significant increase in water consumption in the CCl_4_ group compared with the control group (*p* < 0.0001). No significant difference was observed between the CCl_4_ group and the groups treated with the aqueous and ethanolic fractions (*p* > 0.05), whereas a highly significant difference was observed between the positive control, in which rats underwent oral administration of Sylimarin, and the CCl_4_ group (*p* < 0.01). Finally, a highly significant reduction in water consumption was noted in the group that underwent oral administration of n-hexane fraction (*p* < 0.0001) (Table 3).

### 2.4. Nigella Sativa Effect on Liver Markers

Measurements of the various liver parameters—ALT, AST, ALP, LDH, and GGT—are shown in Table 4. The negative control group, which underwent intraperitoneal injection of CCl_4_ at a dose of 1 mL/kg/week, showed a very highly significant increase in ALT, AST, and ALP compared with the control group (*p* < 0.0001). However, treatment of the different groups with the different fractions of black cumin, at a dose of 250 mg/kg, resulted in a highly significant reduction in the various parameters.

Regarding ALT, there was a highly significant reduction in ALT levels in the Sylimarin and aqueous group (*p* < 0.0001), a highly significant decrease in the ethanolic group (*p* < 0.001), and a statistically highly significant difference in the n-hexane group (*p* < 0.01) compared with the CCl_4_ group. Also, no statistically significant difference was observed between the ethanolic and CCl_4_ groups (*p* > 0.05) (Table 4).

Additionally, CCl_4_ injection showed a statistically highly significant increase in AST levels compared with the control group (*p* < 0.0001). Furthermore, a highly significant difference was noted between the positive control and n-hexane group. Also, there was a highly significant difference between the groups treated, respectively, with Sylimarin and the n-hexane fraction compared with the CCl_4_ group (*p* < 0.0001). There was also a highly significant difference between the groups treated with the aqueous and ethanolic fractions on one hand and the negative control on the other hand (*p* < 0.001), while no significant difference was observed in the methanolic group (*p* > 0.05) (Table 4).

For ALP, the n-hexane and silymarin groups exhibited a highly significant reduction compared to CCl_4_ (*p* < 0.0001), while the ethanolic group showed a significant difference from the negative control (*p* < 0.05). Finally, for the other fractions tested, no significant difference was recorded in comparison with the negative control (*p* > 0.05). For GGT and LDH levels, we note that there is no significant change in the CCl_4_ group compared with the other groups (*p* > 0.05).

### 2.5. NS Fraction Effects on Direct and Total Bilirubin

Table 4 shows the effect of administering different fractions of black cumin at a dose of 250 mg/kg on total and direct bilirubin. At the end of the experiment, there was a statistically highly significant increase in direct and total bilirubin in the CCl_4_ group compared with the control group (*p* < 0.0001), suggesting liver dysfunction. Treatment of rats with 250 mg/kg of the various *NS* fractions resulted in a statistically highly significant attenuation of direct bilirubin levels, compared with the CCl_4_ group (*p* < 0.0001). Meanwhile, no variation was observed in total bilirubin levels, except for the Sylimarin-treated group, which showed a highly significant difference from the CCl_4_ group (*p* < 0.001) (Table 4).

### 2.6. Effects of NS Fractions on Lipid Profile

In this study, the per os administration of the different *NS* fractions on hepatic metabolism was assessed (Table 4). After CCl_4_ injection at a dose of 1 mL/kg/week, there was a highly significant increase in triglycerides, compared with the control group (*p* < 0.001). However, the administration of Sylimarin (50 mg/kg) or the aqueous or n-hexane fraction at a dose of 250 mg/kg to the various CCl_4_-intoxicated rats, showed a highly significant statistical difference with the CCl_4_ group (*p* < 0.0001); meanwhile, the methanolic and ethanolic fractions showed no significant difference with the CCl_4_ group (*p* > 0.05). Regarding the LDL levels, the results obtained showed no significant difference between the different treatments (*p* > 0.05). Moreover, the results recorded for HDL levels showed a highly significant increase for the groups treated with Sylimarin (50 mg/kg) and the methanolic fraction (250 mg/kg) (*p* < 0.001), compared to the control; however, no statistically significant difference was observed in the other groups (*p* > 0.05) (Table 4).

### 2.7. Impact of Nigella Sativa Fractions on Urinary Parameters

Analysis of the various urinary parameters in the control group showed Creatinine levels of 522.5 mg/L, albumin 17.7 µg/L, urea 37.9 g/L, and uric acid 135.5 mg/L. However, CCl_4_ administration in the negative control group and the other groups showed no significant difference in uric acid and creatinine levels compared with the values recorded in the control group (*p* > 0.05) (Table 4). A statistically highly significant increase in albumin levels was observed between the CCl_4_ and control groups (*p* < 0.01). Rats treated with Sylimarin and the aqueous fraction, at a dose of 50 mg/kg and 250 mg/kg, respectively, showed a highly significant reduction in albumin level compared with the CCl_4_ group (*p* < 0.001). On the other hand, a highly significant reduction in albumin levels was recorded in the groups that underwent *per os* administration of the methanolic, ethanolic, and n-hexane fractions at a dose of 250 mg/kg; compared with the CCl_4_ group (*p* < 0.0001) (Table 4).

### 2.8. Effect of NS Fractions on Plasma Renal Markers

Measurements of renal markers such as creatinine, urea, and uric acid are shown in Table 4. In the group that underwent only intraperitoneal *(i.p.)* administration at a weekly dose of 1 mL/kg of CCl_4_, a very highly significant rise in creatinine and urea levels was observed, compared with the control group (*p* < 0.0001). Meanwhile, no significant difference was observed in uric acid between the different groups (*p* > 0.05). Treatment with Sylimarin (50 mg/kg) or with the different fractions of *NS* (250 mg/kg) resulted in a significant improvement in creatinine and urea levels in CCl_4_-intoxicated rats compared with the negative control (CCl_4_) (Table 4).

### 2.9. Effect of NS Fractions on Electrolytes

Plasma and urine electrolyte concentrations are shown in Table 5. The results show that CCl_4_ injection did not affect the concentration of the various electrolytes compared with the control group (*p* > 0.05). It is also noted that there was no effect on the concentration of the various electrolytes for the group treated with Sylimarin (50 mg/kg) and also for the groups treated with the various fractions (250 mg/kg) compared with the CCl_4_ negative control (*p* > 0.05) (Table 5).

### 2.10. Effects of NS Fractions on Hepatic Glycogen

**Figure 1:**
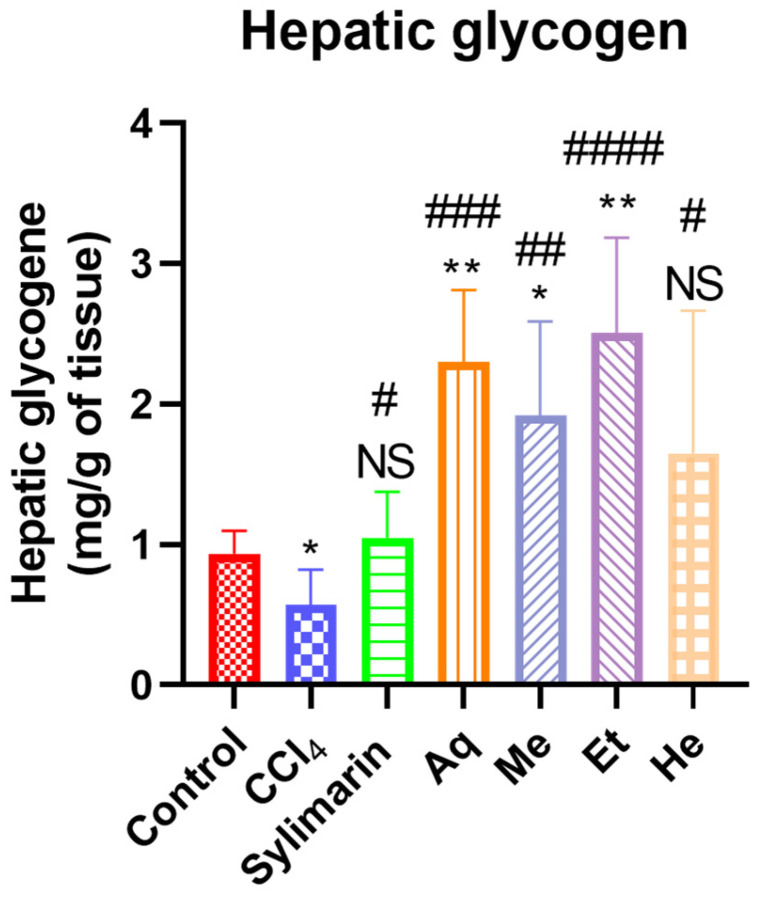
Effects of *Nigella sativa* fractions (250 mg/kg) on hepatic glycogen levels in CCl_4_-intoxicated Wistar rats (n = 6). Ns: Not significant; * *p* < 0.05, ** *p* < 0.01 compared to the control group; # *p* < 0.05, ## *p* < 0.01, ### *p* < 0.001, #### *p* < 0.0001 compared to the CCl_4_ group; values are presented as mean ± sem.

The effects of *per os* administration of aqueous, methanolic, ethanolic, and n-hexane fractions on hepatic glycogen in rats intoxicated with CCl_4_ are shown in Figure 1. In the control group, the level of hepatic glycogen was 0.94 mg/g of tissue. Injection of CCl_4_ alone in rats resulted in a significant decrease in hepatic glycogen levels (0.56 mg/g of tissue) compared to the control group (*p* < 0.05). Regarding the group orally administered 50 mg/kg/day of Sylimarin, a significant increase in glycogen level was observed compared to the negative control group (*p* < 0.05). As for the groups intoxicated with CCl_4_ (1 mL/kg/week) and treated with different fractions of *NS* (250 mg/kg), a highly significant increase in glycogen level was noted in rats treated with the aqueous fraction (*p* < 0.001), a very significant increase in the group treated with the methanolic fraction (*p* < 0.01), a statistically very highly significant increase in those treated with the ethanolic fraction (*p* < 0.0001), and a significant difference in the group treated with the n-hexane fraction (*p* < 0.05), compared to the negative control (Figure 1).

### 2.11. Effects of NS Fractions on Malondialdehyde (MDA)

**Figure 2:**
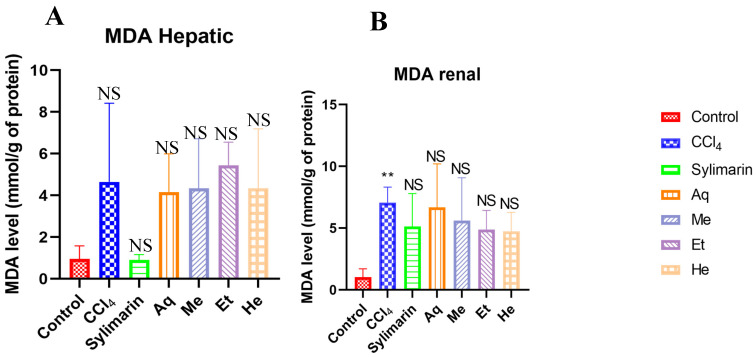
Effect of *Nigella sativa* fractions (250 mg/kg) on hepatic and renal Malondialdehyde levels in CCl_4_-intoxicated rats (n = 6). Ns: Not significant; ** *p* < 0.01 compared to the control group; Ns: Not significant compared to the CCl_4_ group; values are presented as mean ± sem.

The results regarding the level of malondialdehyde in the liver and kidney are represented in Figure 2. It is noted that there is no significant difference between the level of hepatic MDA and the CCl_4_ group (*p* > 0.05). Regarding renal MDA, a significant difference is observed between the control and the CCl_4_ group (*p* < 0.05) on one hand, while no significant difference was mentioned between the groups treated with either Sylimarin or the different fractions of *NS* (*p* > 0.05) (Figure 2).

### 2.12. Molecular Docking Study

#### 2.12.1. Interaction with CYP P450 3E1

**Figure 3:**
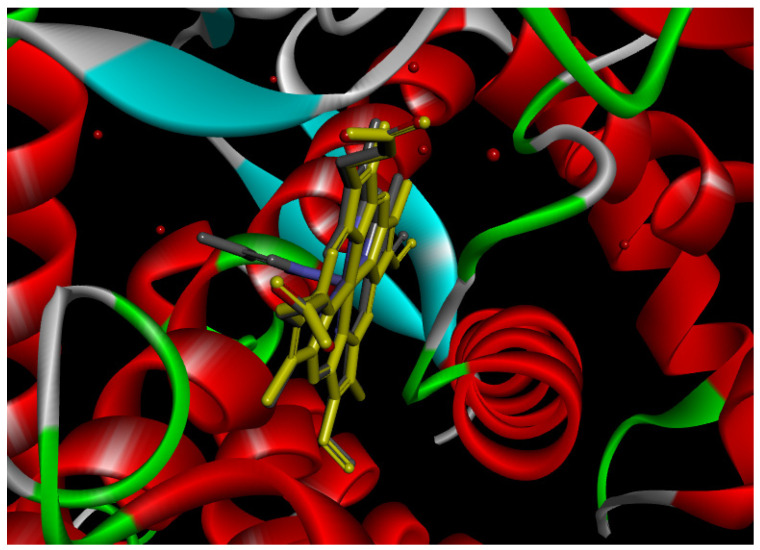
Superposition of the origin Protoporphyrin Ix-Containing Fe native ligand and the best re-docked position of Protoporphyrin Ix-Containing Fe (Yellow) in the binding pocket of human CYP P450 3E1 (3E4E).

The docking procedure was initiated by re-docking with a native ligand (Protoporphyrin Ix Containing Fe) to the binding site of the crystal structure of the Human cytochrome P450 2E1 (3E4E) [36]. The native ligand showed interaction with different residues of the protein. The ligand showed electrostatic bond interaction; also, the protein showed conventional hydrogen bonds. Furthermore, the ligand showed interaction with several protein residues via Van der Waals forces. The re-docking of the native ligand indicated similar conformational poses, hence similar interactions with amino acids, where the co-crystallized Protoporphyrin Ix-Containing Fe is located (Figure 3).

Docking with the native ligand showed a binding affinity of −233.97 Kcal/mol which was the lowest value compared to the tested compounds. Analysis of the docking results indicated that silymarin, a positive control used in the assessed in vivo study, yielded a binding energy of −115.14 Kcal/mol. Sylimarin was predicted to be able to interact with receptor residue, while it could interact via Van der Waals bonds with amino acid receptor residues (Table 6). Additionally, catechin, a compound found in *NS* fractions, was found to have a low binding energy (−104.05 Kcal/mol) which is comparable to that obtained by the native ligand and the positive control. This molecule was found to interact via a hydrogen bond with Cys 437 and by Van der Waals bonds with several residues. Furthermore, rutin (−87.43 Kcal/mol), salicylic acid (−82.78 Kcal/mol), vanillic acid (−80.07 Kcal/mol), and gallic acid (−70.61) were also found to have relatively high binding energies, the 2D representations of the best-docked poses of the studied ligands in the active pocked of human CYP P450 3E1 showed that these compounds were able to interact with a multitude of amino acids that forms the pocket site of the active site (Table 6).

The recorded values were found to be relatively comparable to that of silymarin used as positive control in in vivo experiments, which suggests that *NS* fractions are rich in different phenolic compounds that, alone or synergistically, lead to an inhibition of CYP3E1 which significantly inhibit the metabolization of CCl_4_ into two hyperreactive elements trichloromethyl radical (CCl_3_•) and the trichloromethyl peroxyl radical (Cl_3_COO•).

Overall, the studied bioactive compounds, as well as Sylimarin used as a positive control, were found to interact with the active site of CYP3E1 with similar amino acid residues as the native ligand, as shown in the 2D representations of the best-docked poses of the studied ligands in the active pocket of human CYP P450 3E1.

#### 2.12.2. Interaction with TNF-α

**Figure 4:**
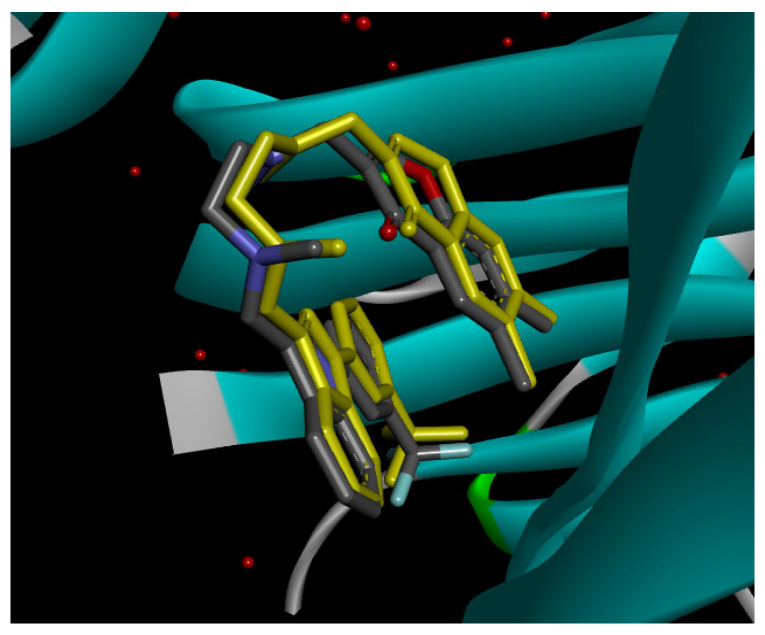
Superposition of the origin SPD 304 native ligand and the best re-docked position of SPD 304 (Yellow) in the binding pocket of TNF-α (2AZ5).

Re-docking process of the TNF-α native ligand SPD 304 showed a perfect superposition profile with the results reported in the literature with the co-crystalized ligand with a binding energy of −114.37 Kcal/mol (Figure 4) [37]. Furthermore, the results indicated that the SPD304 native ligand binds with TNF-α (2AZ5) by hydrogen bonds with Leu 120 and Gly 121, and by Van der Waals with Tyr 119, Leu 120, Gly 121, Tyr 59, Ser60, Tyr 119, Tyr 119, and Gly 121, which represents the catalytic site of the enzyme [38,39].

Analysis of the docking results performed using IGEMDock indicated that rutin is characterized by the lowest binding energy with a value of −122.88 Kcal/mol. Also, it was found that this molecule interacts with the enzyme active site by hydrogen bonds and by Van der Waals bonds with several amino acids, followed by the native ligand that showed a binding energy of −114.37 Kcal/mol. Furthermore, Sylimarin ranked in third place with a binding affinity of −98.94 Kcal/mol; this molecule was found to interact with amino acid residue Gly 121 by a hydrogen bond and with receptor residues that form the catalytic site on the enzyme by Van der Waals. However, catechin was characterized by an energy of −20.24 Kcal/mol, and no hydrogen bond was detected, while a Van der Waals bond was detected (Table 7). On the other hand, gallic acid, vanillic acid, and salicylic acid showed relatively higher binding energy compared to the other compounds with binding energies of −72.29, −62.65, and −61.48 Kcal/mol, respectively. Also, it was noted that these compounds could interact with different amino acid residues of TNF-α (Table 7).

Overall, the different bioactive compounds of *NS* fractions showed promising results on the inhibition of TNF-α which could be considered an important therapeutic strategy to manage inflammatory conditions induced by CCl_4_ intoxication and to exert hepato- and nephroprotection.

#### 2.12.3. Interaction with COX-2

**Figure 5:**
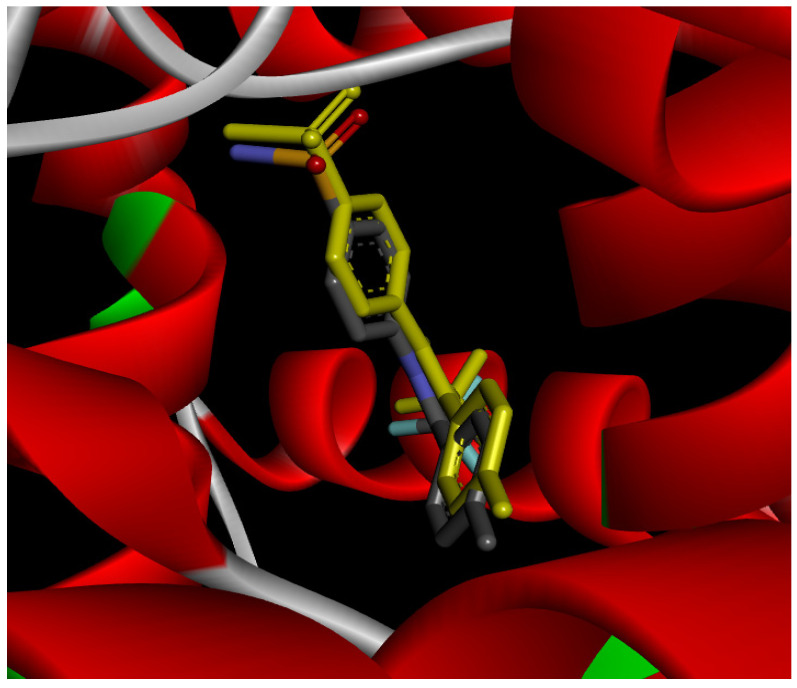
Superposition of the origin Celecoxib and the best re-docked position of Celecoxib (Yellow) in the binding pocket of COX-2 (3LN1).

Re-docking of celecoxib with co-crystalized COX-2 showed an existing superposition with the native ligand, as mentioned in previous studies (Figure 5) [40]. The native ligand showed interaction by hydrogen bonds. Moreover, the native ligand showed interaction with active site residues through Van der Waals bonds.

The results of molecular docking analysis indicated that Celecoxib showed the lowest binding energy of −141.78 Kcal/mol. Moreover, catechin, a compound found in *NS* fractions, showed low binding energy comparable to that obtained by the native ligand that was equal to −114.72 Kcal/mol (Table 8). Catechin has been shown to have conventional hydrogen bonds. Also, it is found to bond with several receptor amino acids by Van der Waals bonds. Additionally, rutin also had a binding energy of −105.08 that was close to the binding energy of the native ligands. Rutin was found to form hydrogen bonds and Van der Waals bonds with receptor residues that form the catalytic site of the enzyme. However, Sylimarin, vanillic acid, and salicylic acid showed binding energies of −94.35, −74.19, and −68.17 Kcal/mol, which were relatively low compared to the native ligand. These mentioned compounds were found to interact with amino acid receptor residues of the COX-2 active site (Table 8).

Overall, the different bioactive compounds of *NS* fractions showed promising results on the inhibition of COX-2 which could be considered an important therapeutic strategy for managing the inflammatory status of liver and kidney tissues.

#### 2.12.4. ADMET Setting Prediction

The different compounds of *Nigella sativa* fractions were subject to pharmacokinetic parameter prediction using (PkCSM) (https://biosig.lab.uq.edu.au/pkcsm/prediction) accessed on 1 May 2024. The obtained results are tabulated in Table 9 below, where it is noted that all compounds exhibited low Caco-2 permeability which suggests limited absorption in the gut. However, the prediction system showed a high predicted permeability percentage through the human intestine which could indicate the passage of the compounds into internal cavities. Moreover, all tested compounds are likely to be substrates for P-Glycoprotein (Table 9). However, rutin showed an ability to inhibit hERG channels which could lead to drug-induced long QT syndrome that is characterized by a prolongation of the QT interval of the electrocardiogram [41].

Additionally, rutin and silymarin were the only compounds with P-Glycoprotein I and II inhibition. Also, the ADMET prediction system indicated that these compounds could have high skin permeability (Table 9). Concerning the volume of distribution, all recorded values indicated a low volume of distribution, indicating a lower concentration in tissues compared to plasma. Furthermore, in silico prediction results showed poor permeability through the blood–brain barrier. Similarly, catechin, rutin, and silymarin are noted to be unable to penetrate the central nervous system (Table 9). On the other hand, silymarin was predicted to be a CYP3A4 substrate, while no compound was mentioned as an inhibitor of CYP P450 enzymes. Finally, the predicted lethal doses at 50 (LD_50_) using *per os* acute toxicity test in rats were found to range between 1.526 and 2.184 mol/kg for all compounds, indicating moderate toxicity levels (Table 9).

## 3. Discussion

Oxidative stress is a process directly linked to the production of reactive oxygen species (ROS). Typically, the radicals formed are neutralized by an intrinsic antioxidant system that detoxifies the body from various ROS produced during biochemical reactions. Despite the existence of a complex intrinsic antioxidant system, the damage from oxidative stress remains unavoidable, which could be a primary cause in the development of several diseases [42]. The hepatoprotective and nephroprotective effects of different *Nigella sativa* fractions (*NS*) were evaluated using the carbon tetrachloride (CCl_4_) model. Upon administration, CCl_4_ is metabolized in the liver specifically by the cytochrome P450 2E1 complex, resulting in the formation of two highly unstable free radicals, trichloromethyl radical (CCl_3_•) and trichloromethyl peroxyl radical (Cl_3_COO•), which are responsible for hepatotoxic and nephrotoxic effects [43]. The existence of these formed radicals leads to various damages within the body, such as lipid peroxidation, release of cytosolic and endoplasmic enzymes, and glomerular and cortical tubular necrosis, indicating damage to the structure and function of the liver and kidneys [44].

The effect induced at the renal and hepatic levels by the administration of CCl_4_ was reflected in the increase in various hepatic markers such as AST (aspartate aminotransferase), ALT (alanine aminotransferase), ALP (alkaline phosphatase), and GGT (γ-glutamyl transferase) [45]. Aromatic and medicinal plants, through their antioxidant properties, can have hepatoprotective and nephroprotective effects by neutralizing free radicals. Black seeds are well-known for their richness in various bioactive compounds such as ferulic acid, gallic acid, quercetin, rutin, and kaempferol, as well as their antioxidant and anti-inflammatory properties [46]. In order to elucidate the hepatoprotective and nephroprotective effects of the various extracted fractions, different hepatic and renal markers were measured.

In the current study, the intraperitoneal injection of CCl_4_ (1 mL/kg/week) significantly decreased the body weight of Wistar rats. However, rats intoxicated with CCl_4_ and treated with 250 mg/kg of various fractions (aqueous, methanolic, ethanolic, and n-hexane extracts) showed an improvement in body weight. Concerning the relative weights of the liver and right kidney, no significant difference was observed between the control group, the CCl_4_ group, and the other treated groups. However, a significant difference was noted in the weight of the left kidney between the group treated with CCl_4_ and the other groups treated with 250 mg/kg of aqueous, methanolic, and n-hexane fraction of *Nigella sativa*.

A significant increase in various hepatic and renal markers was recorded in the CCl_4_ group compared to the control group treated with distilled water. On the other hand, after treatment with Sylimarin (50 mg/kg) or with *NS* fractions (250 mg/kg), a significant decrease was noted in the different groups intoxicated with CCl_4_. These results demonstrated that the nephrotoxic and hepatotoxic effects can be attenuated by administering *Nigella sativa* seed fractions. The results reported by Adam et al. (2016) are consistent with those described in our study. It was clearly demonstrated that the hydroalcoholic extract of *Nigella sativa* (methanol/water) led to a decrease in various hepatic markers such as ALT, AST, LDH, and ALP after acetaminophen-induced intoxication. It was also mentioned in the same study that treatment of TIB-73 liver cells with acetaminophen induced an increase in ROS formation, while co-treatment with *Nigella sativa* seed extract improved cell viability and blocked ROS formation. This extract was noted to be capable of reducing the level of malondialdehyde [47]. In another study, the aqueous extract was mentioned to significantly reduce various hepatic markers in rats intoxicated with CCl_4_. Administration of this extract also led to an increase in intrinsic antioxidant system elements and inhibition of MDA release [48].

Our results align with those of Al-Ghamdi (2003), where per os administration of the aqueous extract at doses of 250 mg/kg and 500 mg/kg led to an improvement in the various parameters studied [49]. Moreover, black seed oil has been proven to have a hepatoprotective effect against various injuries induced by CCl_4_. These results were confirmed by histopathological studies, where it was mentioned that treatment with this oil showed normal hepatocytes without any signs of inflammation [33,50]. Furthermore, black seed oil (800 mg/kg) provided protection against the damage caused by paracetamol, as reflected in the hepatic profile of treated Long Evans rats [51]. Additionally, *NS* seeds also improved various measured parameters in streptozotocin-intoxicated rats, with clear effects observed histologically [52].

Regarding the nephroprotective effect, it was mentioned that the administration of *NS* oil (5 mL/kg) resulted in an improvement in urinary parameters with an increase in the urine volume of treated rats [53]. Treatments of intoxicated rats with powdered cisplatin, ethanolic extract, or fixed oils led to a reduction in creatinine, urea, and uric acid levels, which was consistent with the results reported in the present study, except for uric acid, where no significant difference was observed compared to the CCl_4_ group [54]. Furthermore, it was demonstrated that thymoquinone, an active ingredient in *NS* and its fixed oil, promotes the formation of intrinsic antioxidant elements, leading to the improvement in oxidative damage induced by cisplatin. *NS* oil, in turn, attenuated the increase in urea and creatinine induced by cisplatin. It has also been reported in the literature that these seeds provide protection against anti-inflammatory drugs such as paracetamol [55]. Similarly, fixed oil tested on intoxicated rats with gentamicin provided nephroprotection, as indicated by the reduction in nephrotoxicity indicators. A synergistic nephroprotective effect was recorded after the combination of black seeds and vitamin C [56]. Likewise, in another study by Hosseinian et al. (2018), the hydroalcoholic extract of *NS* and vitamin E protected against cisplatin-induced damage, with no significant difference noted in malondialdehyde (MDA) levels between the different treated groups, which is in accordance with the results reported in our study [53].

Furthermore, the assessed molecular docking study indicated that the different biocompounds tested in silico showed moderate to high binding affinity with the three target proteins CYP P450 3E1, TNF-α, and COX-2. Also, these bioactive compounds were found to interact with the amino acid residues of the protein active pocket. These findings suggest that *Nigella sativa* fractions could be endowed with a high capacity for managing inflammatory conditions in the kidney and liver by the inhibition of COX-2 and TNF-α. Also, *NS* fractions could play an important hepatoprotective and nephroprotective role by inhibiting CYP P450 3E1. The bioactive compounds in *NS*, such as thymoquinone, thymol, gallic acid, quercetin, and α-hederin, are known to exert their effects through multiple pathways [16]. Thymoquinone, for instance, has been shown to scavenge free radicals and reduce oxidative stress by increasing the activity of antioxidant enzymes like superoxide dismutase, glutathione, and catalase [57]. This action likely mitigates lipid peroxidation and cellular damage induced by CCl_4_. Furthermore, *NS* is known for its anti-inflammatory properties, particularly through the inhibition of TNF-α and COX-2 [58]. By modulating these inflammatory mediators, *NS* could prevent the deterioration of tissue damage in the liver and kidneys [59]. The molecular docking results in this study further confirmed that bioactive compounds from *NS* exhibit strong binding affinities for these proteins, suggesting that NS can inhibit their activity and contribute to its overall protective effects. The inhibition of CYP P450 3E1 by NS is a crucial mechanism by which the generation of toxic metabolites from CCl_4_ is minimized. By blocking the metabolism of CCl_4_ *into* its highly reactive intermediates (CCl_3_• and Cl_3_COO•), NS could reduce the oxidative stress and subsequent organ damage associated with CCl_4_ exposure.

In the present study, the *per os* administration of different *Nigella sativa* fractions (at a dose of 250 mg/kg), obtained using a Soxhlet apparatus, showed a great ability to restore various liver enzymes to normal levels against the harmful effects induced by CCl_4_ injection. These effects could be attributed to the various bioactive compounds identified by HPLC. Hence, these findings still have numerous limitations for translating animal experiments toward humans; first, animal experiments often present methodological bias, including the absence of uniform standards for the presentation of animal data. Furthermore, animal models do not always faithfully reproduce human pathophysiology, which is one of the challenges of the translation of bench research to clinical practice [60]. Also, animals are generally young, without comorbidities, and do not undergo the multiple interventions that human patients often receive. The timing, route of administration, and formulation of treatments can pose specific problems in animal studies. Additionally, animal experiments often use small sample sizes, which can lead to an overestimation of the effects observed, which misleads researchers when selecting only positive results and ignoring negative ones, and this phenomenon is called “Optimism bias” [60]. Among the suggested solutions to reduce bias is the creation of a registration system for animal experiments similar to that of clinical trials. High caution must be taken when applying animal results to human clinical treatments [61]. Different approaches are adopted to circumvent these differences, such as work at the genetic and molecular levels, and cellular levels in order to understand the link between animals and humans [62].

## 4. Materials and Methods

### 4.1. Chemicals

Carbon tetrachloride was purchased from Sigma-Aldrich Chemicals (CCl_4_ ≥ 99.5%), St Louis, MO, USA. Silymarin from Sigma-Aldrich (95%), Belgium. Thiobarbituric acid (TBA ≥ 98%) and trichloroacetic acid (TCA ≥99.0%) were purchased from Sigma Chemicals, Germany. Methanol (CH_3_OH, ≥99.8%), ethanol (C_2_H_5_OH, ≥99.8%), dichloromethane (CH_2_Cl_2_ ≥ 99.8%), and hexane (C_6_H_14_, ≥97.0%) were purchased from Sigma-Aldrich (St. Louis, MO, USA). Standard diagnostic kits were supplied by Abbott Laboratories and were compatible with the ARCHITECT ci8200 analyzer. For hepatic enzymes Alanine Aminotransferase (ALT) Ref 7D56-21, Aspartate Aminotransferase (AST) Ref 7D81-21, Alkaline Phosphatase (ALP) Ref 7D55, Lactate Dehydrogenase (LDH) Ref 2P56, γ-glutamyl Transferase (GGT) Ref 7D65, Albumin Ref 7D53, Triglycerides (TG) Ref 7D74-21, Cholesterol Ref 7D62-21, Low-density lipoprotein (LDL) Ref 1E31-20, High-density lipoprotein (HDL) Ref 3K33-21, creatinine Ref 3L81, urea Ref 7D75, uric acid Ref 3P39-21, total bilirubin (BT) Ref 6L45-21, direct bilirubin (BD) Ref 8G63-21, and electrolyte levels (Calcium, sodium, potassium, chlore). All reagents used in this work were of high quality and analytical grade.

### 4.2. Plant Material

*Nigella sativa* seeds were purchased from a local market in Oujda, Morocco. A specimen was deposited at the University Mohammed Premier Herbarium under the number HUMPOM471.

### 4.3. Extraction Procedure

*Nigella sativa* (*NS*) seeds were ground to a fine powder using an electric grinder before extraction. The Soxhlet apparatus consists of a glass extractor, placed between a round-bottomed flask and a condenser at the top. Inside the glass thimble holder, the seed powder is placed. Next, 100 g of *NS* were extracted with Hexane for fatty acid removal for about eight hours. After each extraction, the residual plant material was air-dried to remove any remaining solvents and transferred to Dichloromethane to obtain a dichloromethane fraction. The remaining plant material was air-dried once more and extracted with ethanol, then methanol, and finally distilled water. Each extraction step was followed by an air-drying process to prepare the plant material for the next solvent. All fractions obtained were filtered through Whatman filter paper to remove any particles and stored at 4 °C for about one week before the start of the experiment.

### 4.4. Qualitative and Semi-Quantitative Analysis of n-Hexane Extract

The NF T60-233 methodology provided by Aïssi et al., (2009) [63] was followed in methyl ester preparation. A gas chromatograph (Shimadzu GC-2010) fitted with a fused-silica capillary column (5% phenyl methyl siloxane, 30 m × 0.25 μm film thickness) and a mass spectrometer detector (GC-MS-QP2010) was used to examine the esterified n-hexane fraction. The carrier gas, helium, was set to a steady pressure of 100 kPa. The oven was preheated to 50 °C for one minute, and then it was gradually raised to 250 °C for one minute at a rate of 10 °C per minute. Injector, transfer line, and ion source temperatures were adjusted to 250 °C, 250 °C, and 200 °C, respectively. Solutions comprising 1 μL of the materials diluted in hexane (50 mg/g) were injected in split mode (50–80), and the GC-MS system was run in scan mode for the qualitative and semiqualitative analyses. With a range of 40–350 a.m.u., mass spectra were acquired at 70 eV (electron impact ionization mode). The rate and solvent delays were 5 s/scan and 4.5 min, respectively. By comparing their MS data with those kept on the National Institute of Standards and Technology (NIST147) computer library, the fatty acid components were identified. Data processing and collection were conducted using LabSolutions (version 2.5) [24].

### 4.5. Qualitative and Quantitative Analysis Using High-Performance Liquid Chromatography (HPLC-UV)

The various fractions (aqueous, MeOH, and EtOH) were prepared at a concentration of 20 mg/mL in order to determine various HPLC profiles. Following that, a filtration using Millipore 20 μm filters was completed. Next, each sample was divided into 20 μL and injected into an Alliance ew2695, C_18_ (250 × 4 mm, 5 μm) reversed-phase column. The high-performance liquid chromatography (HPLC) system was coupled to a PDA Waters 2996 UV detector, operating within a wavelength range of 210–400 nm. The analysis was performed using a linear gradient, with the UV detector set at 254 nm for compound detection. The HPLC analysis was carried out with 80% water in acetic acid for 20 min, 100% methanol for 25 min, and a flow rate of 1 mL/min in between. The HPLC program Empower (version 3) was used to examine the peak regions and heights. Many compounds, including gallic acid, vanillic acid, naringenin, rutin, catechin, kaempferol, vanillin, ferulic acid, and salicylic acid, were used in relation to the HPLC profile. The various analytical standards were prepared in DMSO (1 mg/mL). The identical process outlined above was used to inject 10 μL into the HPLC-UV. The concentration of phenolic compounds was measured using each standard’s calibration curve. Three duplicates of the analysis were performed. Every calibration curve exhibited strong linearity, r^2^ > 0.99 [35].

### 4.6. Animals

Housing and husbandry conditions were monitored daily by a qualified technician. Bedding was changed twice per week, and cages were cleaned regularly to ensure hygienic and stress-free conditions. Animals were observed for signs of discomfort or distress, and care was taken to minimize any handling-related stress.

In this experiment, strains of Wistar rats, both male and female, weighing between 150 and 300 g and aged between 8 and 10 weeks, were used. The animals were acquired from the biology department’s animal facility at Mohammed First University’s Faculty of Sciences in Oujda, Morocco. The rats (n = 42) were housed individually in standard plastic cages for acclimatization. The rats were provided with ad libitum access to water and a standard rodent chow (pellets) supplied by Provimac SA, Meknes, Morocco, and were maintained under standard laboratory conditions, including a constant temperature of 21 ± 2 °C, relative humidity (40–60%), and a 12 h light/dark cycle. The acclimatization period lasted one week, allowing the animals to adapt to their new environment before the commencement of the experimental procedures. This careful attention to housing and acclimatization conditions is critical for minimizing stress and ensuring the validity of the experimental outcomes.

The application of pain management complied with ethical standards. To avoid possible pain during delicate procedures, ether was utilized as an anesthetic. Animals were never given needless pain or suffering during the course of the experiment. The administration of anesthesia was conducted in a well-ventilated fume hood to ensure the safety of the researchers and minimize animal exposure to excessive vapors. International ethical standards for the treatment and use of laboratory animals were closely followed by the experimental procedures. The 3Rs (Replacement, Reduction, and Refinement) were also adhered to in the study design, which ensured the ethical treatment of animals by minimizing their numbers while maximizing the reliability and scientific value of the data collected.

### 4.7. Ethical Statement

The hepatoprotective and nephroprotective study was assessed according to the US National Institutes of Health’s international guidelines for the use and care of laboratory animals [64]. The Vice Dean of Scientific Research at the Faculty of Sciences, University Mohammed First of Oujda, has confirmed adherence to all animal experimentation requirements. This is documented in a signed and stamped certificate, verifying that each animal test was conducted by the internationally accepted Guide for the Care and Use of Laboratory Animals (Supplementary file).

### 4.8. Experimental Design

In the experimental study, carbon tetrachloride (CCl_4_) was used due to its well-documented ability to induce oxidative stress and cellular damage, making it a reliable model for studying liver and kidney injuries. CCl_4_ metabolism generates reactive intermediates which disrupt lipid metabolism, cause lipid peroxidation, and lead to cellular damage, steatosis, apoptosis, and fibrosis. Also, its nephrotoxic effects are evidenced by changes such as hydropic degeneration of renal tubules. These mechanisms mimic human liver and kidney pathologies, making CCl_4_ a valuable tool for exploring therapeutic interventions and understanding toxicological processes [65]. Following a week of adaptation, the animals were split into seven groups at random, each with six rats (3 ♂/3 ♀). The CCl_4_ group and the control group (CG) were given 10 milliliters per kilogram of distilled water. Rats were given 50 mg/kg of sylimarin as a positive control. Silymarin, derived from the milk thistle plant (Silybum marianum), is a well-established hepatoprotective agent recognized for its pharmacological efficacy in both preclinical and clinical settings [66]. This compound has demonstrated significant antioxidant properties, including the ability to scavenge free radicals, prevent lipid peroxidation, and enhance the body’s antioxidant defenses [67]. The concentration of 50 mg/kg was selected based on its documented effectiveness in previous studies and its common use as a benchmark in experiments assessing hepatoprotective agents. Silymarin’s well-characterized mechanism of action and reliable results in various studies make it an appropriate and repeatable positive control for evaluating the protective effects of treatments against liver damage [67,68]. The other groups received 250 mg/kg of each black cumin fraction. Following a week of each pre-treatment, CCl_4_ (solubilized in *v*/*v* olive oil) was administered intraperitoneally (*i.p.)* to the animals once a week for 15 days (days 7 and 14) at a dose of 1 mL/kg.

This dosage was selected based on its efficacy in inducing hepatotoxicity and nephrotoxicity while minimizing excessive toxicity that could lead to high mortality. The frequency of administration was designed to mimic a relevant exposure scenario that allows for the assessment of both acute and chronic effects of CCl_4_ on liver and kidney function. Following 0, 7, 9, 11, and 15 days of treatment, body weights were recorded. For two weeks, every animal received treatment and was monitored every day. Additionally, all animals’ water intake and urine volume were measured using metabolic cages at the end of treatment [69].

### 4.9. Blood Collection

In a fume hood laboratory, ether was used in a tiny jar to induce anesthesia in all animals twelve hours after the last CCl_4_ injection. All animals were then sacrificed. Samples of blood were drawn from the artery in the abdomen and put into dry blood collection tubes. The plasma was then separated by centrifuging the blood at 4 °C for 10 min and 3000 rpm. Until biochemical analysis, plasma was kept at −20 °C.

### 4.10. Evaluation of Liver and Kidney Homogenates

In order to prepare homogenates (20% *w*/*v* in 0.1 mM phosphate buffer, pH 7.4), liver and kidney tissues from every group that was sacrificed were weighed and stored. These homogenates were stored at −20 °C until the liver glycogen content and malondialdehyde (MDA) levels were measured. MDA concentrations were found using the TBA reaction method to measure hepatic and renal lipid peroxidation. Following homogenate preparation, 1 mL of the homogenate supernatant was combined with 1 mL of TBA reagent, which is made up of 15% TCA and 0.67% *w*/*v* TBA dissolved in 0.25 N HCl. After 30 min in a boiling water bath, the reaction mixture was centrifuged for 5 min at 4000 rpm. The molar extinction coefficient of 1.56 × 10^5^ M^−1^·cm^−1^ was used to calculate the MDA quantities after the absorbance was measured at 535 nm. The findings are presented as nanomoles of MDA per milligram of kidney or liver tissue (nmoL/mg) [70], respectively. In contrast, liver glycogen was measured using the method outlined by Ong et al. [71]. A quantity of liver (0.3 to 0.5 g) was mixed with 2 mL of 30% KOH solution to create the liver homogenate. For 30 min, the tissue was boiled at 100 °C. The mixture was then mixed with 4 milliliters of 95% ethanol to precipitate the glycogen. The purified glycogen was then dissolved by adding 1 milliliter of distilled water. Five milliliters of the Anthrone reagent were added in order to calculate the caloric content of hepatic glycogen.

The results, which were expressed as mg/kg tissue, were obtained by measuring absorbance at 625 nm. The following formula was used to estimate the impact of oral administration of black cumin fractions on the variation in liver and kidney weight ratios:

$$Organ\;weight\;ratio\;(\%)=\frac{Organ\;weight}{Animal\;weight}\times 100$$

The body weight variation was calculated according to the following formula:

$$Body\;Weight\;Variation\;(\%)=\frac{Final\;body\;weight-Initial\;body\;weight}{Initial\;body\;weight}\times 100$$

where

Final Body Weight is the weight of the subject at the 7th or 15th day of the experiment;

Initial Body Weight is the weight of the subject at the start of the experiment.

### 4.11. Biochemical Analyses

Hepatic, lipid, and renal markers were measured using the ARCHITECT ci8200 analyzer developed by Abbott Laboratories, an advanced clinical chemistry and immunoassay system designed for high-volume laboratories, according to manufacturer guidelines, including calibration and quality control procedures. The analyzer processes raw data from sample measurements to generate quantitative results for each tested analyte: Alanine Aminotransferase (ALT), Aspartate Aminotransferase (AST), Alkaline Phosphatase (ALP), Lactate Dehydrogenase (LDH), γ-glutamyl Transferase (GGT), Triglycerides (TG), low-density lipoprotein (LDL), high-density lipoprotein (HDL), creatinine, urea, uric acid, albumin, total bilirubin (BT), direct bilirubin (BD), and electrolyte levels.

### 4.12. Molecular Docking Analysis

The bioactive compounds of *Nigella sativa* fractions found in high quantities such as catechin, rutin, vanillic acid, salicylic acid, and gallic acid were selected to perform a molecular docking study using iGEMDOCK version 2.1 in order to evaluate the possible interaction with target proteins involved in inflammation and oxidative stress induced by CCl_4_ intoxication which allows a better understanding of the molecular mechanisms of action underlying hepatoprotective and nephroprotective effect of *NS* fractions against induced CCl_4_ toxicity.

### 4.13. Ligand Preparation

Target compound 3D structures were retrieved from PubChem database in SDF format, catechin (CID: 9064), Rutin (CID: 5280805), Vanillic acid (CID: 8468), Gallic acid (CID: 370), Salicylic acid (CID: 338), and Silymarin (CID: 5213) and then were converted into pdb format using Chem3D 16.0. The 3D formats of target proteins, CYP P450 3E1 (PDB ID: 3E4E), TFN-a (PDB ID: 2AZ5), and COX-2 (PDB ID: 3LN1) [72,73], were downloaded from the Protein Data Bank (PDB). All files were downloaded in pdb format.

The visualization of the ligand–protein interaction was performed using BIOVIA Discovery Studio 2021.

### 4.14. Evaluation of ADMET Settings

An in silico approach was adopted to assess ADMET settings for six main bioactive compounds found in *Nigella sativa* fractions, which provides valuable data on the pharmacokinetic drug properties of each compound. The ADMET analysis was performed using ^(PkCSM)^ (http://biosig.unimelb.edu.au/pkcsm/) accessed on 1 May 2024 [74].

### 4.15. Statistical Analysis

The results were subject to statistical descriptive analysis and variance analysis (ANOVA), using Graphpad prism 9 for MacOS, and the comparison between the different means was carried out using the Tukey test with a probability threshold of 5%.

## 5. Conclusions

Based on our results, we conclude that the fractions of *Nigella sativa* (*NS*) have no adverse effects. Furthermore, these different fractions significantly protect against the renal and hepatic toxicity caused by CCl_4_. They could intercept the free radicals released by CCl_4_, acting as a free radical scavenger. The hepatoprotective and nephroprotective effects obtained could be attributed to the various bioactive compounds present in each fraction and their capacity to interact with numerous target proteins (TNF-α, COX-2, and CYP P450 3E1) in order to alleviate inflammatory reactions. Nevertheless, this research has limitations, including that the obtained mechanism from molecular docking is preliminary and more experiments are needed for the validation of these results (e.g., enzyme inhibition assays or Western blotting). Moreover, our study only focused on a single toxin and acute exposure model, which might not accurately represent chronic or multifactorial organ damage. Also, the specific bioactive compounds that caused the observed effects were not isolated or tested separately, leaving their relative contributions unclear. Furthermore, isolating the active biocompounds in *NS* fractions, confirming their target interactions in in vitro and in vivo tests, and investigating their effectiveness in chronic or multi-organ toxicity models should be the top priorities of future study. Finally, comparative studies should be assessed with standard hepatoprotective/nephroprotective agents for better contextualization of the therapeutic potential of this plant’s bioactive compounds.

## Figures and Tables

**Table 1:** Per os administration effect of *Nigella sativa* (250 mg/kg) on the body weight of CCl_4_-intoxicated Wistar rats.

Groups	Average Body Weight (g)					Body Weight Gain (g)		Body Weight Variation (%)	
	Day 0	7th Day	9th Day	12th Day	15th Day	7th Day	15th Day	7th Day	15th Day
Control	186.3 ± 8.75	202.3 ± 8.07	204.8 ± 5.52	209.2 ± 5.29	211.1 ± 6.87	16	24.8	8.58	11.8
CCl_4_	173 ± 7.62	189.1 ± 6.06	180 ± 6.12 ****	183.3 ± 6.17 ****	185.6 ± 7.85 ****	16.1	12.6	9.30	6.78
Sylimarin	185.3 ± 12.79	200.7 ± 9.01	192.8 ± 10.83 ^Ns^	200 ± 11.22 ^Ns#^	200.3 ± 13.06 ^Ns^	15.4	15	8.31	7.48
Aqueous extract (Aq)	186.3 ± 4.04	202.97 ± 7.44	191.97 ± 8 ^Ns^	190.7 ± 8 **^Ns^	204.47 ± 6.10 ^Ns##^	16.67	18.17	8.94	8.88
Methanolic extract (Me)	180.12 ± 9.75	184.40 ± 9.12	196.005 ± 6.99 ^Ns#^	205.6 ± 8.3 ^Ns###^	188.66 ± 7.01 ***^Ns^	4.28	8.54	2.37	4.52
Ethanolic extract (Et)	172.1 ± 5.59	193.96 ± 10.27	186.3 ± 11.01 **^Ns^	210.7 ± 10.1 ^Ns####^	209.35 ± 10.12 ^Ns###^	21.86	15.39	12.7	7.35
n-hexane extract (He)	183.46 ± 11.14	214.08 ± 10.71	205.82 ± 9.55 ^Ns####^	215.8 ± 13.1 ^Ns####^	215.10 ± 15.03 ^Ns####^	30.62	31.64	16.69	14.70

^Ns^: not significant; ** *p* < 0.01, *** *p* < 0.001, **** *p* < 0.0001 comparison with control group; ^#^ *p* < 0.05, ^##^ *p* < 0.01, ^###^ *p* < 0.001, ^####^ *p* < 0.0001 comparison with CCl_4_ group; values are presented as mean ± SEM.

**Table 2:** *Per os* administration of different *Nigella sativa* fractions (250 mg/kg) on liver and kidney weight ratios in CCl_4_-intoxicated Wistar rats.

Groups	Liver Ratio (%)	Kidney Ratio (%)	
		Left kidney	Right kidney
Control	2.734 ± 0.062	0.378 ± 0.026	0.359 ± 0.014
CCl_4_	4.351 ± 0.109 *	0.376 ± 0.021 ^Ns^	0.362 ± 0.009 ^Ns^
Sylimarin	3.572 ± 0.162	0.347 ± 0.009	0.359 ± 0.008
Aq	3.35 ± 0.17	0.269 ± 0.004	0.264 ± 0.003
Me	3.62 ± 0.14	0.308 ± 0.006	0.320 ± 0.005
Et	3.93 ± 0.33	0.307 ± 0.006	0.319 ± 0.025
He	4.13 ± 0.39	0.32 ± 0.03	0.334 ± 0.027

^Ns^: not significant; * *p* < 0.05 comparison with control group; values are presented as mean ± sem.

**Table 3:** *Per os* administration of different *Nigella sativa* fractions (250 mg/kg) on water consumption and urine volume in CCl_4_-intoxicated Wistar rats.

Groups	Water Consumption (mL/24 h)	Urine Volume (mL/24 h)
Control	19.16 ± 1.20	6.83 ± 1.50
CCl_4_	41.00 ± 4.57 ****	10.42 ± 1.10 *
Sylimarin	29.16 ± 1.80 * ^#^	10.83 ± 1.41 ** ^Ns^
Aq	35.00± 3.53 *** ^Ns^	7.5 ± 0.35 ^Ns^
Me	28.75 ± 1.87 * ^#^	10.62 ± 0.93 *^Ns^
Et	32.50 ± 1.88 **^Ns^	7.25 ± 0.69 ^Ns #^
He	21.80 ± 5.43 ^Ns ####^	12.40 ± 1.37 *** ^Ns^

^Ns^: not significant; * *p* < 0.05, ** *p* < 0.01, *** *p* < 0.001, **** *p* < 0.0001 comparison with control group; ^#^ *p* < 0.05, ^####^ *p* < 0.0001 comparison with CCl_4_ group; values are presented as mean ± sem.

**Table 4:** Biochemical and lipid profile effects of *Nigella sativa* fractions in CCl_4_-intoxicated rats.

Parameter		Groups						
		Control Group	CCl_4_ Group	Sylimarin Group	Aq	Me	Et	He
**Liver parameter** **s**	Alanine Aminotransferase (ALT)	51.20 ± 7.87	1014.33 ± 68.18 ****	202.20 ± 61.32 ^ns, ####^	245.00 ± 32.52 ^ns, ####^	378.5 ± 50.69 ^ns, ###^	639.80 ± 82.25 ***^, ns^	539.20 ± 92.48 **^, ##^
	Aspartate Aminotransferase (AST)	179.83 ± 36.79	1188.83 ± 75.48 ****	287.00 ± 58.24 ^ns, ####^	743.00 ± 23.13 ***^, ##^	870.00 ± 5.85 ****^, ns^	643.20 ± 73.43 **^, ###^	600.80 ± 70.16 **^, ####^
	Lactate Dehydrogenase (LDH)	486.83 ± 105.26	653.17 ± 151.96 ^ns^	754.17 ± 169.63 ^ns^	1366.00 ± 85.60 ^ns^	1995.00 ± 0.00 ^ns^	1466.80 ± 211.42 ^ns^	1785.2 ± 85.65 ^ns^
	Alkaline Phosphatase (ALP)	171.33 ± 21.36	512.67 ± 16.96 ****	159.00 ± 31.84 ^ns, ####^	596.00 ± 25.45 ****^, ns^	381.00 ± 0.41 ***^, ns^	337.80 ± 18.43 **^, ##^	247.40 ± 22.02 ^ns, ####^
	Gamma-Glutamyl Transferase (GGT)	4.66 ± 0.45	9.83 ± 3.42 ^ns^	9.60 ± 2.50 ^ns^	4.00 ± 0.00 ^ns^	4.5 ± 0.07 ^ns^	4.00 ± 0.00 ^ns^	4.00 ± 0.00 ^ns^
	Total Bilirubin	1.27 ± 0.17	4.45 ± 0.61 ****	1.64 ± 0.18 ^ns, ###^	5.15 ± 0.51 ****^, ns^	5.70 ± 0.19 ****^, ns^	3.33 ± 0.17 **^, ns^	2.75 ± 0.41 ^ns, #^
	Direct Bilirubin	0.67 ± 0.26	3.83 ± 0.51 ****	1.00 ± 0.00 ^ns, ####^	1.50 ± 0.096 ^ns, ####^	1.00 ± 0.00 ^ns, ####^	1.00 ± 0.00 ^ns, ####^	1.00 ± 0.00 ^ns, ####^
**Urinary Parameters**	Urea	37.97 ± 2.08	23.66 ± 2.76 ****	30.622 ± 2.15 ^ns^	42.40 ± 0.00 ^ns, ####^	42.40 ± 0.00 ^ns, ####^	42.29 ± 0.04 ^ns, ####^	41.99 ± 0.22 ^ns, ####^
	Creatinine	522.59 ± 54.82	374.33 ± 32.13 ^ns^	409.12 ± 49.41 ^ns^	512.775 ± 8.81 ^ns^	381.46 ± 5.98 ^ns^	436.24 ± 20.58 ^ns^	452.022 ± 39.62 ^ns^
	Uric Acid	135.58 ± 24.56	87.92 ± 6.85 ^ns^	130.13 ± 12.76 ^ns^	102.15 ± 6.74 ^ns^	117.75 ± 1.54 ^ns^	106.08 ± 10.21 ^ns^	115.22 ± 19.73 ^ns^
	Albumin	17.67 ± 1.63	58.33 ± 13.29 **	19.40 ± 2.75 ^ns. ##^	25.00 ± 0.27 ^ns, ##^	13.50 ± 0.07 ^ns, ###^	12.00 ± 2.04 ^ns, ###^	6.00 ± 0.27 ^ns, ####^
**Lipidic Parameters**	Low-Density Lipoprotein (LDL)	0.224 ± 0.01	0.280 ± 0.007 ^ns^	0.216 ± 0.019 ^ns^	0.095 ± 0.009 ^ns^	0.140 ± 0.016 ^ns^	0.074 ± 0.012 ^ns^	0.073 ± 0.012 ^ns^
	High-Density Lipoprotein (HDL)	0.248 ± 0.005	0.182 ± 0.002 **	0.248 ± 0.007 ^ns, ##^	0.135 ± 0.002 ****^, ns^	0.120 ± 0.005 ****^, ##^	0.226 ± 0.013 ^ns^	0.156 ± 0.009 ****^, ns^
	Triglycerides (TG)	0.44 ± 0.008	0.725 ± 0.053 ***	0.40 ± 0.05 ^ns, ####^	0.37 ± 0.003 ^ns, ####^	0.58 ± 0.02 ^ns^	0.57 ± 0.02 ^ns^	0.41 ± 0.01 ^ns, ####^

Aq: Aqueous fraction; Et: Ethanolic fraction; He: Hexanic fraction; Me: Methanolic fraction. ^Ns^: not significant; ** *p* < 0.01, *** *p* < 0.001, **** *p*< 0.05 in comparison with control group, ^#^ *p* < 0.05, ^##^ *p* < 0.01, ^###^ *p* < 0.001, ^####^ *p* in comparison with CCl_4_ group. values are presented as mean ± SEM (n = 6).

**Table 5:** Effect of *NS* fractions on electrolyte levels of CCl_4_-intoxicated Wistar rats.

Electrolytes	Groups	Control	CCl_4_	Sylimarin	Aqueous Fraction	Methanolic Fraction	Ethanolic Fraction	n-Hexane Fraction
Na^+^(mM)	Plasma	178.83 ± 8.64	175.83 ± 7.01 ^Ns^	172.60 ± 5.01	135 ± 0.06	132 ± 0.82	131.25 ± 2.52	127.4 ± 3.37
	Urine	95.2 ± 16.39	143.8 ± 15.28 ^Ns^	86.6 ± 11.41	240 ± 0.00	239.8 ± 0.14	476 ± 84.43	175 ± 34.88
Ca^2+^(mM)	Plasma	78.6 ± 2.67	89.6 ± 2.64 ^Ns^	82.16 ± 2.57	96.35 ± 0.05	84.1 ± 0.12	78.4 ± 4.6	75.76 ± 3.15
	Urine	199.17 ± 12.72	126.50 ± 20.75 ^Ns^	184 ± 20.55	82.5 ± 7.55	83 ± 2.31	101.25 ± 8.68	86.8 ± 9.44
K^+^(mM)	Plasma	4.08 ± 0.23	5 ± 0.68 ^Ns^	4.3 ± 0.26	4.35 ± 0.06	4.35 ± 0.03	4.94 ± 0.39	5.46 ± 0.33
	Urine	177 ± 10.82	132.42 ± 21.33 ^Ns^	155.96 ± 14.5	240 ± 8.16	150.15 ± 2.86	182.83 ± 10.8	134.9 ± 11.60
Cl^-^(mM)	Plasma	77.6 ± 8.84	83.3 ± 5.4 ^Ns^	78 ± 4.08	101.5 ± 0.2	105.5 ± 0.34	94.25 ± 2.38	97.5 ± 1.7
	Urine	110.83 ± 7.42	58.83 ± 15.72 ^Ns^	96.60 ± 7.38	77.50 ± 7.82	58 ± 1.22	108.75 ± 12.42	98.8 ± 10.70

^Ns^: not significant; Comparison with the control group; Values are presented as mean ± SEM.

**Table 6:** Binding energies of bioactive compounds and involved amino acid residues of CYP P450 3E1.

Molecules	Binding Energy (Kcal/mol)	Involved Receptor Residues	2D-Representations of the Best-Docked Poses of the Studied Ligands in the Active Pocket of Human CYP P450 3E1 (3E4E).
Catechin	−104.05	H-Ala299, Thr 303, Thr 304, Cys 437, Ala 438, Gly 439, Ala 443, Glu 446, V-Ile 115, Ile 115, Ala 299, Gly 300, Thr 303, Thr 303, Thr 307, Arg 435, Val 436, Cys 437, Cys 437, Ala 438, Gly 439, Glu 440, Leu 442,	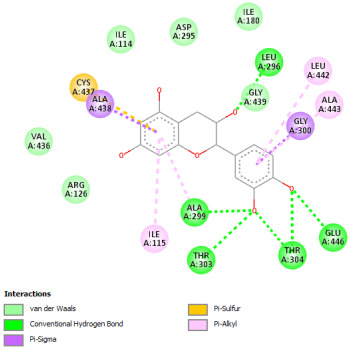
Sylimarin	−115.14	H-Arg-126; Thr303; Thr307; Gln358; Leu363; Cys 437; Ala 438; V- Arg 100; Ile115; Ile115; Ala-299; Thr-303; Thr-303; Thr-307; Leu363; Leu-363; Val-364; Leu-368; Pro-429; Phe-430; Phe-430; Arg-435; Arg-435; Val-436; Cys-437; Cys-437; Ala-438; Gly-439; Phe-430, Arg 435, Arg 435, Val 436, Cys 437, Cys 437, Ala 438, Gly 439	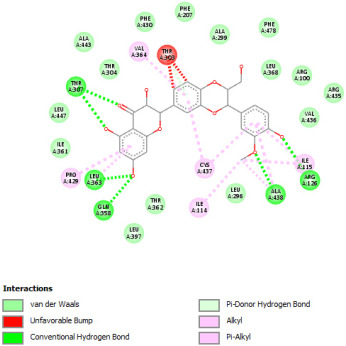
Rutin	−87.43	H-Arg126, Leu 130, Cys 437, Gly 439, Glu 440, Gly441, Leu 442, Gly 439, Glu 440, Gly 441, Leu 442, V-Leu 130, Leu 130, Arg 435, Val 436, Cys 437, Cys 437, Ala 438, Gly 439, Glu 440, Glu 440, Gly441, Leu 442	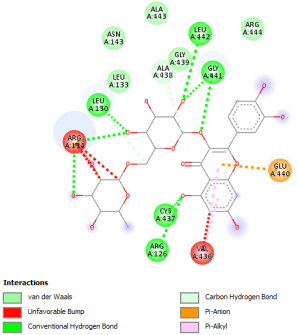
Gallic acid	−70.61	H-Ala 229; Thr 303; Thr 304; Cys437, Glu446; V-Ala 299; Gly 300; Thr 303; Thr 303; Phe 430; Cys 437; Ala-438; Gly-439; Glu 440, Leu 442	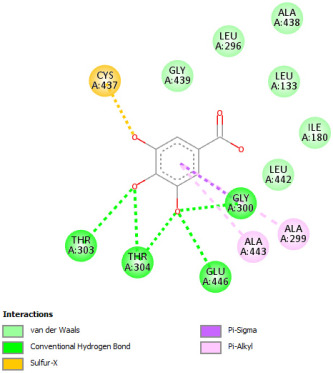
Salicylic acid	−82.78	E-Arg 100, Arg 435, H-Arg 100, Trp 122, Arg 126, Arg 435, Ala 438, V- Arg 100, Ile 115, Ile 115, Leu 368, Arg 435, Arg 435, Val 436, Cys 437, Cys 437, Ala 438, Gly 439	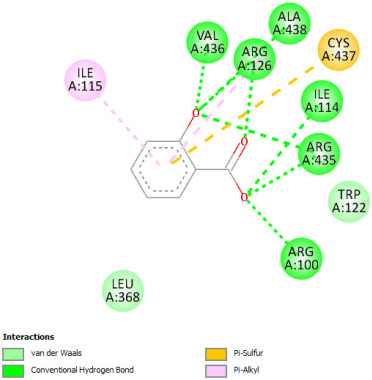
Vanillic acid	−80.07	E-Arg-100; Arg-435; H-Arg-100; Trp-122; Arg-126; Arg-435; Cys-437; Ala-438; V-Arg-100; Ile-115; Ile-115; Ala-299; Leu-368; Arg-435; Arg-435; Val-436; Cys-437; Cys-437; Ala-438; Gly-439	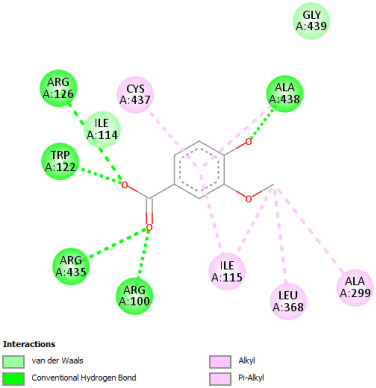
Protoporphyrin Ix Containing Fe	−233.97	E-Arg 100, His 370, Arg 435, H-Arg 100, Trp 122, Arg 126, His 370, Ser 431, Arg 435, Cys 437, V-Arg 100, Ile 115, Ile 115, Ala 299, Gly 300, Thr 303, Thr 303, Thr 307, Leu 363, Leu 363, Val 364, Leu 368, Pro 429, Phe 430, Arg 435, Arg 435, Val 436, Cys 437, Cys 437, Ala 438, Gly 439, Glu440, Leu 442	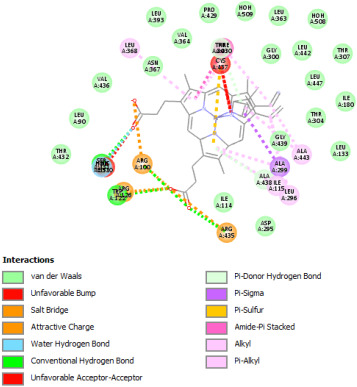

**Table 7:** Binding energies of bioactive compounds and involved amino acid residues of TNF-α (2AZ5).

Molecules	Binding Energy (Kcal/mol)	Involved Receptor Residues	2D-Representations of the Best-Docked Poses of the Studied Ligands in the Active Pocket of TNF-α (2AZ5)
Rutin	−122.88	(H- Ser 60; Leu 120; Gly 121; Ser 60; Leu 120; Tyr 151); (V-Tyr 59; Ser 60; Gln 61; Tyr 119; Tyr 119; Leu 120; Gly 121; Leu 57; Tyr 59; Tyr 59; Ser 60; Tyr 119; Leu 120; Gly 121; Tyr 151; Leu 55)	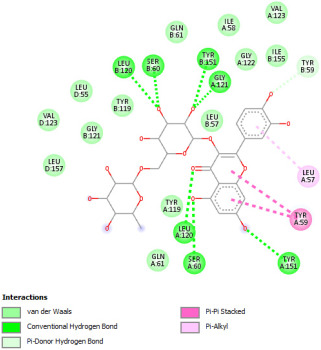
Sylimarin	−98.94	(H-Gly 121; Gly 121; Tyr 151; Leu 55); (V-Tyr 59; Tyr 119; Tyr 119; Leu 120; Gly 121; Leu 57; Tyr 59; Tyr 59; Ser 60; Tyr 119; Leu 120; Gly 121; Tyr 151; Leu 55)	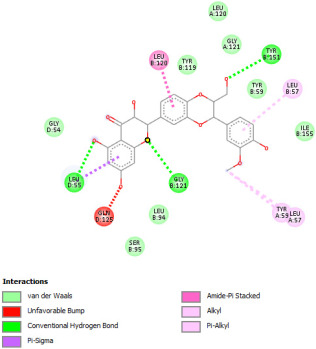
Catechin	−90.24	(H- Ser 60; Gln 61; Ser 60; Tyr 151); (V-Tyr 59; Ser 60; Gln 61; Tyr 119; Tyr 119; Leu 120; Gly 121; Tyr 59; Tyr 59; Ser 60; Tyr 119; Leu 120; Gly 121; Tyr 151)	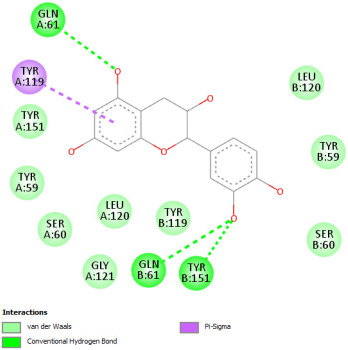
Gallic acid	−72.29	(H-Leu 120; Gly 121; Ser 60; Leu 120; Tyr 151); (V- Tyr 119; Tyr 119; Leu 120; Gly 121; Tyr 59; Tyr 59; Ser 60; Tyr 119; Leu 120; Gly 121; Tyr 151)	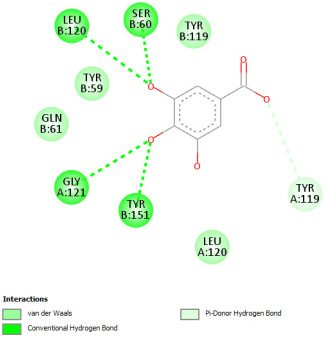
Vanillic acid	−62.65	(H-Gly 121; Ser 60; Leu 120; Tyr 151); (V-Tyr 59; Tyr 119; Tyr 119; Leu 120; Gly 121; Leu 57; Tyr 59; Tyr 59; Ser 60; Tyr 119; Leu 120; Gly 121; Tyr 151)	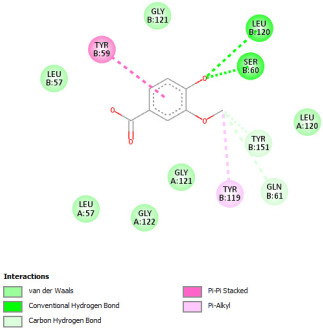
Salicylic acid	−61.48	(H- Gly 121; Ser 60; Leu 120; Tyr 151); (V-Tyr 119; Tyr 119; Leu 120; Gly 121; Leu 57; Tyr 59; Tyr 59; Ser 60; Tyr 119; Leu 120; Gly 121; Tyr 151)	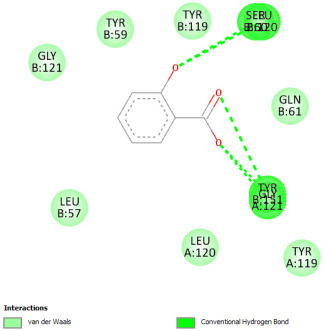
SPD304 (native inhibitor of TNF a)	−114.37	(H-Leu 120; Gly 121); (V-Tyr 119; Leu120; Gly121; Tyr 59; Ser 60; Tyr 119; Tyr 119; Gly 121)	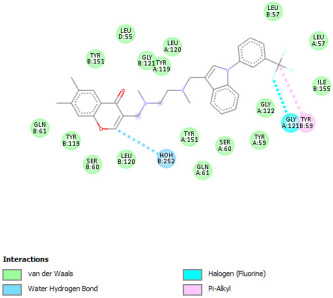

**Table 8:** Binding energies of bioactive compounds and involved amino acid residues of COX-2 (3LN1).

Molecules	Binding Energy (Kcal/mol)	Involved Receptor Residues	2D-Representations of the Best-Docked Poses of the Studied Ligands in the Active Pocket of COX-2 (3LN1)
Celecoxib (native inhibitor of COX-2)	−141.78	(H-His 75, Gln 178, Leu 338, Ser339, Tyr341, Arg499, Phe 504, V- His75, Val 335, Leu 338, Ser 339, Tyr 341, Phe 367, Tyr 371, Trp 373, Arg 499, Arg 499, Phe 504, Val 509, Val 509, Gly 512, Ala 513, Ser 516)	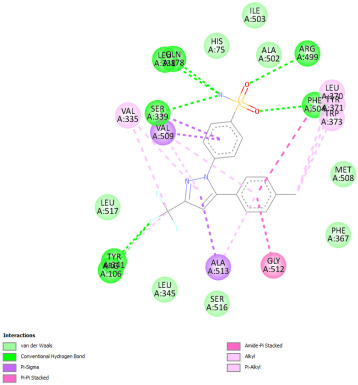
Catechin	−114.72	(H-Phe 196, Tyr 371, Phe 515, Phe 516, Ser 516, Leu 520, V-Phe 191, Thr 192, Phe 196, Phe 196, Val 335, Phe 367, Tyr 371, Ser 516)	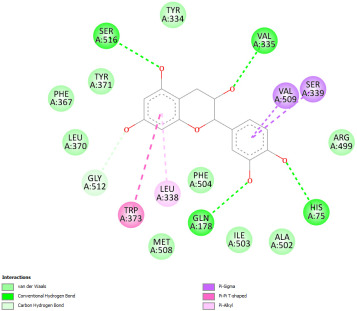
Rutin	−105.08	H-His 75, Gln 178, Val 335, Leu 338, Ser 339, Ser 516, V- His75, Val 335, Leu 338, Ser 339, Tyr 341, Arg 499, Phe 504, Val 509, Val 509, Gly 512, Ala 513	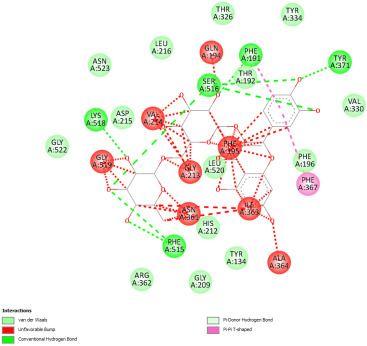
Sylimarin	−94.35	H-His 75, Pro 498, V-ASN72, ASN 72, His 75, Pro 498, Pro 498, Arg 499, Arg 499, Pro 500, Gly 505, Glu 506, Glu 506	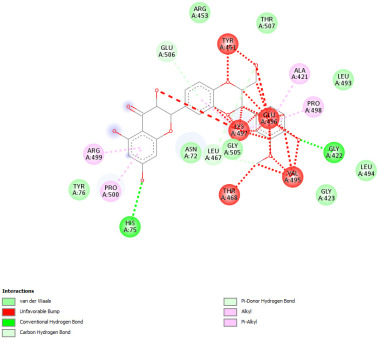
Gallic acid	−69.01	H-His75, Gln 178, Ser 339, Tyr 341, Arg 499, V- His 75, Val 335, Leu 338, Ser 339, Tyr 341, Arg 499, Arg 499, Phe 504, Val 509, Val 509, Ala 513	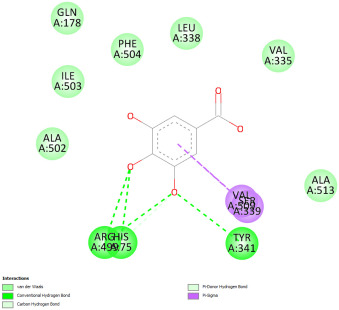
Vanillic acid	−74.19	H-His 75, Phe 504, V- His 75, Val 335, Leu 338, Leu 338, ser 339, Tyr 341, Trp 373, Arg 499, Arg 499, Phe504, Gly 505, Val 509, Val 509, Gly 512, Ala 513	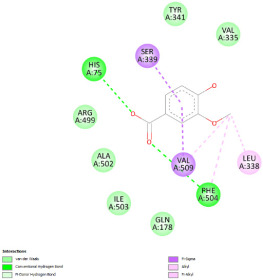
Salicylic acid	−68.17	H-His 75, Ser 339, Tyr 341, Arg 499, V- His 75, Val 335, Leu 338, Leu 338, Ser 339, Tyr 341, Arg 499, Arg 499, Phe 504, Gly 505, Val 509, Val 509	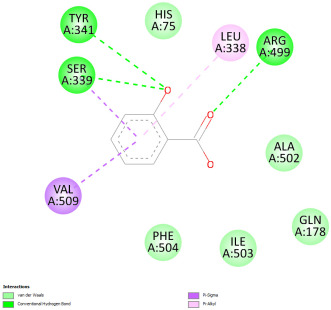

**Table 9:** In silico ADMET properties were identified in different *Nigella sativa* fractions.

		Catechin	Rutin	Gallic Acid	Salicylic Acid	Sylimarin	Vanillic Acid
**Absorption**	Water solubility (log mol/L)	−2.808	−3.327	−0.723	−0.895	−4.304	−0.992
	Caco2 permeability (log Papp in 10^−6^ cm/s)	−0.38	−0.791	−0.467	1.173	−0.363	0.199
	Intestinal absorption (human) (% Absorbed)	71.562	28.495	50.311	74.883	74.315	75.448
	Skin Permeability (log Kp)	−3.603	−2.737	−3.084	−2.868	−2.921	−2.941
	P-glycoprotein substrate (Yes/No)	Yes	Yes	Yes	Yes	YES	Yes
	P-glycoprotein I inhibitor	No	Yes	No	No	Yes	No
	P-glycoprotein II inhibitor	No	Yes	No	No	Yes	No
**Distribution**	VDss (human) (log L/kg)	−0.79	−1.597	−1.078	−0.784	−1.327	−0.907
	Fraction unbound (human) (Fu)	0.326	0.419	0.565	0.496	0.142	0.496
	BBB permeability (log BB)	−0.905	−2.215	−0.93	−0.283	−1.481	−0.295
	CNS permeability (log PS)	−3.146	−4.842	−2.816	−2.437	−3.384	−2.601
**Metabolism**	CYP2D6 substrate (Yes/No)	No	No	No	No	No	No
	CYP3A4 substrate (Yes/No)	No	Yes	No	No	Yes	No
	CYP1A2 inhibitior (Yes/No)	No	No	No	No	No	No
	CYP2C19 inhibitior (Yes/No)	No	No	No	No	No	No
	CYP2C9 inhibitior (Yes/No)	No	No	No	No	No	No
	CYP2D6 inhibitior (Yes/No)	No	No	No	No	No	No
	CYP3A4 inhibitior (Yes/No)	No	No	No	No	No	No
	CYP2D6 substrate (Yes/No)	No	No	No	No	No	No
	CYP3A4 substrate (Yes/No)	No	No	No	No	No	No
	CYP1A2 inhibitior (Yes/No)	No	No	No	No	No	No
**Excretion**	Total Clearance (log ml/min/kg)	0.215	0.187	0.55	0.625	−0.092	0.626
	Renal OCT2 substrate (Yes/No)	No	No	No	No	No	No
**Toxicity**	AMES toxicity (Yes/No)	Yes	No	No	No	No	No
	Max. tolerated dose (human) (log mg/kg/day)	0.956	0.375	1.404	1.541	0.307	1.404
	hERG I inhibitor (Yes/No)	No	No	No	No	No	No
	hERG II inhibitor (Yes/No)	No	Yes	No	No	Yes	No
	Oral Rat Acute Toxicity (LD50) (mol/kg)	2.101	1.526	1.872	2.022	2.184	2.004
	Oral Rat Chronic Toxicity (LOAEL) (log mg/kg_bw/day)	2.076	2.231	1.499	2.875	2.593	2.827
	Hepatotoxicity (Yes/No)	No	No	No	No	No	No
	Skin Sensitisation (Yes/No)	No	No	No	No	No	No
	* T.Pyriformis * toxicity (log ug/L)	0.464	0.285	−0.071	−0.133	0.291	0.028
	Minnow toxicity (log mM)	2.249	4.442	2.918	2.23	1.335	2.183

## Data Availability

The original contributions presented in this study are included in the article. Further inquiries can be directed to the corresponding authors.

## Supplementary Materials

The following supporting information can be downloaded at: https://www.mdpi.com/article/10.3390/ph18081147/s1.
