# Supplementary material for: Harnessing the Hepatoprotective and Nephroprotective Potential of Nigella sativa Fractions via per os Administration in CCl4-Intoxicated Wistar Rats: A Mixed Approach
Source: Pharmaceuticals (Basel). 2025 Aug 1;18(8):1147. doi: 10.3390/ph18081147 (PMC12389490; doi:10.3390/ph18081147)
Supplement: Supplementary file 1 [file pharmaceuticals-18-01147-s001.zip › pharmaceuticals-3509298-supplementary.pdf]

Statement Regarding Ethical Oversight of Animal Research at faculty of sciences Oujda

As a vice dean of Scientific research at the faculty of sciences Oujda we are totally aware of the concerns being raised regarding the oversight of the Ethical Committee in our institution's animal research practices. While it is true that our institution does not have a formal Ethical Committee in place. We would like to assure the scientific community and the public that the care and use of laboratory animals in our research endeavors are conducted in strict accordance with the international guidelines outlined by the US National Institutes of Health (NIH), where the researcher has to:

- Emphasize the importance of maintaining high standards of animal welfare and minimizing discomfort, pain, and distress experienced by laboratory animals.
- Provide recommendations for appropriate housing conditions, including space requirements, temperature, humidity, and lighting, to ensure the well-being of animals.
- Offer guidance on the design and conduct of experimental procedures to minimize potential harm to animals while still achieving scientific objectives.
- Recommend that personnel involved in animal care and research receive appropriate training and possess the necessary qualifications to ensure the treatment of animals and the integrity of scientific research.
- Assuring that the principles of replacement, reduction, and refinement (the 3Rs) to minimize animal use and suffering are performed.

Our researchers and staff undergo extensive training on these guidelines, and we maintain rigorous internal protocols to ensure compliance with these standards.

Furthermore, it is important to note that **Mohammed Dalli , Nour Elhouda Daoudi , Salah-eddine Azizi 1 , Mohammed Roubi , Ilyass Alami Merrouni , Faiza Souna , Mohammed Choukri** and **Nadia Gseyra** involved in the research entitled **"Harnessing the Hepatoprotective and Nephroprotective Potential of Nigella Sativa Fractions via Per os Administration in CCl_4-Intoxicated Wistar Rats: A mixed approach"** presented a comprehensive plan demonstrating that the animals involved did not suffer during the course of the experiments.

We understand the importance of transparency and accountability in animal research, and we welcome any inquiries or requests for further information regarding our animal care and use protocols. Our institution is committed to upholding the highest standards of integrity and responsibility in all our scientific endeavors.

Pr. Khalid Chaabane

Vice dean of Scientific Research at the Faculty of Sciences Oujda

Université Mohammed Premier Faculté des Sciences Oujda

Le Vice-Doyen
Chargé de a Recherche
Scientifique
Khalid CHAABANE

08/07/24
